# Endoscopic Ultrasound-Guided Gastroenterostomy for Gastric Outlet Obstruction: A Comparison of Duodenal Versus Jejunal Placement on Patient Outcomes

**DOI:** 10.1055/a-2913-6628

**Published:** 2026-07-23

**Authors:** Rishad Khan, Ryan J. Law, Chinmay Guralwar, Jacob Bauss, Jad P. AbiMansour, Garima Suman, Samuel Han, Eric J. Vargas, Barham K. Abu Dayyeh, Navtej Buttar, Louis-Michel Wong Kee Song, Andrew C. Storm, Vinay Chandrasekhara

**Affiliations:** 1 Division of Gastroenterology and Hepatology 6915 Mayo ClinicRochesterMinnesotaUnited States; 2 Division of Gastroenterology and Hepatology 2129 University of CalgaryCalgaryAlbertaCanada; 3 Department of Radiology 6915 Mayo ClinicRochesterMinnesotaUnited States; 4 Division of Gastroenterology and Hepatology 22494 Cedars-Sinai Medical CenterLos AngelesCaliforniaUnited States

**Keywords:** endoscopic ultrasonography, intervention EUS, quality and logistical aspects, performance and complications

## Abstract

**Background and Aims** EUS-guided gastroenterostomy (EUS-GE) is an effective option to palliate symptoms of gastric outlet obstruction (GOO). While the jejunum is conventionally targeted (EUS-GJ), targeting the duodenum (EUS-GD) may offer clinical advantages. Outcomes were compared for EUS-GJ and EUS-GD.

**Methods** Adult patients who underwent EUS-GE for GOO between 2020 and 2024 were included and categorized as an EUS-GD if the stent entered the transverse or ascending duodenum or an EUS-GJ if the stent entered more distally. The primary outcome was clinical success (soft diet tolerance at 7 days). Secondary outcomes included adverse events (AEs), stent misdeployment, and unplanned endoscopic reintervention. Baseline demographic variables and outcomes were compared with statistical significance at *p* < 0.05.

**Results** Of the 139 included patients (median age 67 years (IQR 61–74); 41% female), 105 underwent EUS-GJ and 34 underwent EUS-GD. Clinical success was 98% for EUS-GJ and 94% for EUS-GD ( *p* = 0.25). The misdeployment rate was 7% for EUS-GJ and 0% for EUS-GD ( *p* = 0.19). The AE rate was 22% for EUS-GJ and 12% for EUS-GD ( *p* = 0.23). There were no significant differences for stent obstruction (EUS-GJ 12% vs. EUS-GD 6%), bleeding (2% vs. 6%), perforation (4% vs. 0%), stent migration (1% vs. 0%), infection (3% vs. 0%), and unplanned reintervention (14% vs. 9%).

**Conclusion** Both EUS-GJ and EUS-GD demonstrate high clinical success with no significant differences in adverse event rates. These data can support intraprocedural decision-making for endoscopists performing EUS-GE.

## Background

Gastric outlet obstruction (GOO) is a debilitating entity leading to malnutrition, anorexia, and worse oncologic outcomes for patients with gastroduodenal and pancreaticobiliary malignancies. 1 While GOO has conventionally been managed by surgical gastrojejunostomy and placement of enteral self-expandable metal stents, endoscopic-ultrasound guided gastroenterostomy (EUS-GE) has recently emerged as a safe and effective option for the palliation of GOO. 2 Under EUS guidance and a cautery-enhanced delivery catheter, a lumen apposing metal stent (LAMS) is deployed to create an anastomosis between the stomach and an adjacent loop of duodenum or jejunum distant to the site of obstruction. 2

EUS-GE has higher clinical success, decreased risk of stent obstruction, and lower reintervention rates compared to luminal stenting. 3 Compared to surgical gastrojejunostomy, EUS-GE has a lower adverse event (AE) rate, shorter interval times to resume oral intake and chemotherapy, and can be performed on patients who are high-risk surgical candidates. 4 While this procedure was initially conceptualized to replicate a surgical gastrojejunostomy, it became apparent that the distal duodenum may also be a suitable target for endoscopic anastomosis. EUS-gastroduodenostomy (EUS-GD) may represent a physiologically distinct approach from surgical or EUS-gastrojejunostomy (EUS-GJ) given the retroperitoneal location of the duodenum, a relatively fixed position compared to the jejunum, and the axis of the anastomosis into the duodenum being perpendicular to the flow of enteric contents. It is unknown, however, whether these differences are clinically meaningful. To address this gap, we evaluated clinical outcomes and AEs for EUS-GD compared to EUS-GJ.

## Methods

Adult patients (age ≥18) who underwent EUS-GE between January 2020 and December 2024 at a large tertiary care referral center were included. Patients were identified through a prospectively maintained procedural database. Patients who had surgically altered luminal anatomy and those who had luminal obstructions at or distal to the fourth portion of the duodenum were excluded. Patients were also excluded if they did not have any postprocedure cross-sectional imaging, as this imaging was used to classify the location of stent placement. Ethical approval was obtained from the Mayo Clinic Institutional Review Board. A small subset of cases ( *n* = 17) included here were part of a prior multicenter study on EUS-GE outcomes. 5

### Procedural Technique

All procedures were performed by experienced pancreaticobiliary endoscopists under general anesthesia. EUS-GE was performed by first advancing a guidewire and nasobiliary catheter past the site of obstruction, instilling fluid through the catheter to distend the target loop of bowel, and then using a cautery-enhanced LAMS to puncture the gastric and small bowel wall. 6 The distal flange is deployed in the small bowel, followed by advancement of a preloaded guidewire and then deployment of the proximal flange in the stomach. Intravenous glucagon is given prior to stent placement to decrease peristalsis. All stents had a 15 mm or 20 mm luminal diameter and a 10 mm length (HotAXIOS, Boston Scientific Corp, Marlborough, United States). Subsequent balloon dilation of the stent lumen to 10 mm–15 mm was performed at the discretion of the endoscopist. The target loop was selected by the endoscopist based on patient anatomy, identification of a endosonographic window, and anticipated future need to access the gastroenteric LAMS to perform biliary intervention. 7

### Data Extraction

**Fig. 1:**
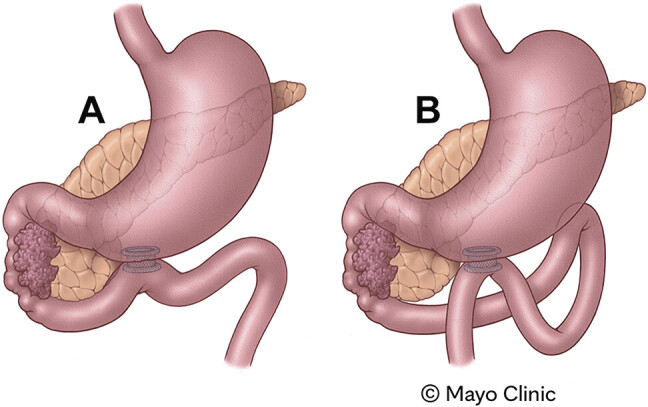
Visual representation of management of gastrointestinal luminal obstruction at the level of the duodenum, with gastroduodenostomy stent (panel A) and gastrojejunostomy stent (panel B). Used with permission of Mayo Foundation for Medical Education and Research; all rights reserved.

Data on baseline demographic variables, etiology of GOO, gastrointestinal symptoms, preprocedure weight and body mass index (BMI) was collected. Stent placement into the duodenum vs. jejunum was evaluated based on postprocedure cross-sectional imaging by a radiologist and endoscopist blinded to outcomes, with any discrepancies resolved by a third investigator. The procedure was categorized as an EUS-GD if the stent was noted to enter the transverse or ascending duodenum, and as an EUS-GJ if the stent entered the inferiorly oriented proximal jejunum or any point distal ( Fig. 1 ). Data regarding AEs, resumption of oral intake, and need for endoscopic reintervention was collected.

### Outcomes

The primary outcome was clinical success, defined as the ability to tolerate at least a soft diet at 7 days post procedure. Secondary outcomes were overall and 30-day AEs, stent misdeployment, and unplanned endoscopic reintervention. AEs included perforation, infection without perforation, bleeding, and stent obstruction or migration causing recurrent GOO symptoms or an unplanned reintervention. 8 Infection without perforation was defined by the presence of fever or leukocytosis post-procedure requiring antibiotics but without imaging findings showing a frank perforation. Misdeployment cases that led to a prespecified AE were categorized as both a misdeployment and the corresponding AE. For example, a misdeployment that was immediately salvaged but associated with postprocedure peritonitis requiring antibiotics would be classified as a misdeployment and a perforation. Misdeployments that resulted in no apparent AE were classified as misdeployment alone. Cases were categorized using a previously published misdeployment classification scheme. 9 Reintervention was defined as the rate of repeat endoscopic intervention for the LAMS. 10 Two post-hoc exploratory analyses were performed: (1) 30-day mortality and change in BMI four weeks post procedure and (2) a time-to-event analysis to compare the duration from the procedure to an AE between the two groups.

### Analysis

Patient characteristics were summarized using counts with percentages and medians with interquartile ranges (IQRs) where appropriate. Baseline variables and outcomes for EUS-GD with EUS-GJ were compared using unpaired *T* test and Mann-Whitney *U* tests for parametric and nonparametric continuous variables, respectively, and Fisher’s exact test for categorical variables.

For the time-to-AE analysis, cumulative probability curves of remaining AE free were generated using the Kaplan–Meier method and compared using the Log-rank test. Cox proportional hazards regression was used to estimate the association between stent location (jejunum versus duodenum) and time to an AE in days, with the estimate presented as a hazard ratio (HR) and associated 95% confidence interval (CI). Patients were censored at death. All statistical tests were two-tailed and a *p* value < 0.05 was considered statistically significant. All analyses were completed in R version 4.5.3.

## Results

There were 162 patients who underwent EUS-GE between January 2020 and December 2024. Fifteen patients were excluded as they did not have postprocedure cross-sectional imaging available. Five patients were excluded due to surgically altered anatomy, and 3 patients excluded due to obstructions distal to the fourth portion of the duodenum. Overall, 139 patients were included in the analysis. The median age was 67 years (IQR 61–74) and 57 (41%) patients were female. All included patients had underlying malignancy, with 70 (50%) having metastatic disease. The most common etiologies of GOO were pancreatic adenocarcinoma ( *n* = 84, 60%), metastatic disease (24, 17%), and duodenal adenocarcinoma ( *n* = 14, 11%). Thirty-three (24%) patients had radiographic evidence of peritoneal carcinomatosis but with no apparent obstruction downstream of the GOO. In all, 39 (28%), 70 (50%) and 63 (45%) patients had baseline symptoms of nausea, postprandial vomiting, and abdominal pain, respectively. The median preprocedure BMI was 22 for the EUS-GJ group (IQR 20–24) and 23 for the EUS-GD group ((IQR 21–29), ( *p* = 0.12). There were no differences between the two groups regarding other baseline variables ( Table 1 ).

**Table 1:** Baseline demographics, clinical variables, outcomes, and adverse events for endoscopic ultrasound-guided gastroduodenostomy compared to gastrojejunostomy.

	EUS GJ ( *n* = 105)	EUS GD ( *n* = 34)	*p*
Baseline variables			
Sex (female)	43 (41%)	14 (41%)	>0.99
Age (median, IQR)	66 (62–74)	67 (63–74)	0.20
Etiology			
Ampullary carcinoma	2 (2%)	0	>0.99
Cholangiocarcinoma	8 (8%)	3 (9%)	0.73
Duodenal adenocarcinoma	12 (11%)	2 (6%)	0.52
Gastric adenocarcinoma	3 (3%)	1 (3%)	>0.99
Metastasis/other	20 (19%)	4 (11)	0.44
Pancreas adenocarcinoma	60 (57%)	24 (69%)	0.23
Metastatic cancer	54 (51%)	16 (47%)	0.7
BMI pre (median, IQR)	22 (20–24)	23 (21–29)	0.12
Symptoms			
Nausea	27 (26%)	12 (35%)	0.28
Vomiting	52 (50%)	18 (53%)	0.84
Abdominal pain	49 (47%)	14 (42%)	0.69
Presence of carcinomatosis	24 (23%)	9 (26%)	0.65
Follow up duration days (median, IQR)	92 (33–193)	72 (26–143)	0.42
**Outcomes and Adverse Events**			
Clinical success	103 (98%)	32 (94%)	0.25
Stent misdeployment	7 (7%)	0	0.19
Adverse event	23 (22%)	4 (12%)	0.22
Obstruction	13 (12%)	2 (6%)	0.36
Bleeding	2 (2%)	2 (6%)	0.25
Perforation	4 (4%)	0	0.57
Migration	1 (1%)	0	>0.99
Infection (without perforation)	3 (3%)	0	>0.99
Unplanned endoscopic reintervention	15 (14%)	3 (9%)	0.56
Exploratory outcomes			
30-day mortality	23 (22%)	12 (35%)	0.17
BMI difference (week 4) (median, IQR)	−1 ([−1.5]–0)	0 ([−2]−1)	0.63

EUS, endoscopic ultrasound; GD, gastroduodenostomy; GJ, gastrojejunostomy; BMI, body mass index; IQR, interquartile range.

A 15 mm diameter LAMS was used in 136 (98%) cases, and a 20 mm diameter LAMS in 3 (2%) cases. Balloon dilation of the LAMS lumen immediately after deployment was performed for 98 (93%) cases in EUS-GJ group and 30 (88%) cases in the EUS-GD group. The median follow-up duration was 92 days (interquartile range [IQR] = 33–193 days) for the EUS-GJ group and 72 days (IQR = 26–143) for the EUS-GD group. Technical success was achieved in all cases.

### Outcomes

For the primary outcome of clinical success, there was no significant difference between the EUS-GJ group ( *n* = 103, 98%) and the EUS-GD group ( *n* = 32, 94%) ( *p* = 0.25) ( Table 1 ). There were seven cases of misdeployment (7%) in the EUS-GJ group, two (2%) of which were not associated with any AE. There were no misdeployments in the EUS-GD group.

**Fig. 2:**
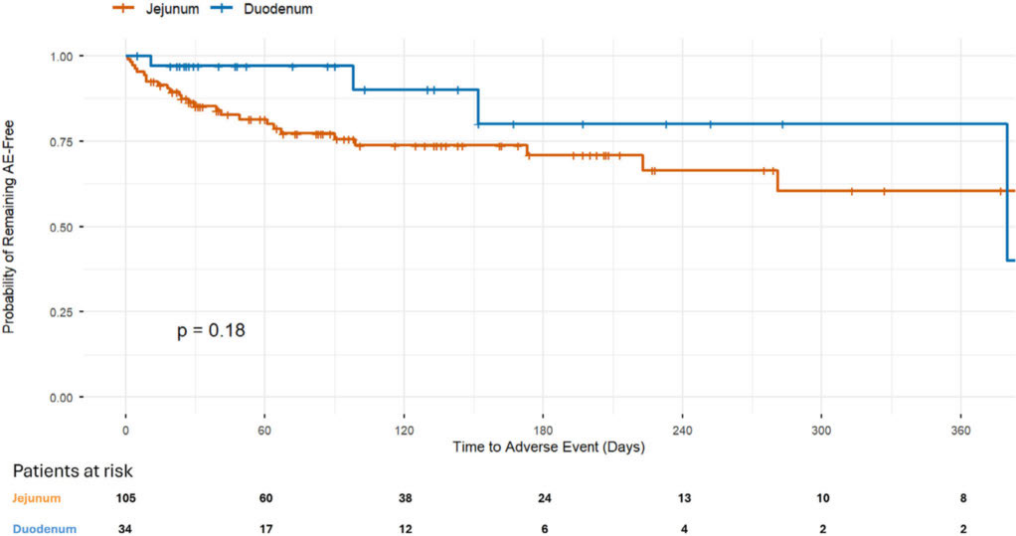
Cumulative probability curves showing the probability of remaining adverse event free for EUS-guided gastroduodenostomy (blue) and EUS-guided gastrojejunostomy (orange). Curves are truncated at 365 days to focus on 1-year outcomes using the Kaplan–Meier method. Patients were censored at death.

There were 23 (22%) AEs in the EUS-GJ group, consisting of obstruction ( *n* = 13, 12%), perforation ( *n* = 4, 4%), infection without perforation ( *n* = 3, 3%), bleeding ( *n* = 2, 2%), and clinically significant migration ( *n* = 1, 1%), and 4 (12%) AEs in the EUS-GD group, consisting of obstruction ( *n* = 2, 6%) and bleeding ( *n* = 2, 6%) with no significant differences between the groups for any AE. There were no cases of death attributable to the procedure. There were no significant differences between groups for 30-day risk of AE ( *n* = 10 [10%] in the EUS-GJ group; *n* = 1 [3%] in the EUS-GD group, *p* = 0.21). There were no significant differences between the two groups in the time-to-AE analysis (HR = 0.49, 95% CI 0.17–1.41, *p* = 0.20). Cumulative probability curves of remaining AE free are shown in Fig. 2 .

There was no significant difference between the two groups for unplanned reintervention (EUS-GJ *n* = 15, 14% vs. EUS-GD *n* = 3, 9%, *p* = 0.56) or 30-day mortality (EUS-GJ group *n* = 23, 22% vs. EUS-GD group *n* = 12, 35%) ( *p* = 0.45). The median BMI at 4-weeks postprocedure was 23 (IQR 21–27) for the EUS-GJ group and 24 (IQR 22–30) for the EUS-GD group. There was no significant difference between the two groups for median BMI change from preprocedure to 4-weeks postprocedure (EUS-GJ −1, 0, IQR [−2]–0) vs. EUS-GD 0, IQR [−2]–1, *p* = 0.63).

### Adverse Events

For the 15 cases of stent obstruction, the median time to obstruction after stent placement was 281 days (IQR = 26–381). Obstruction occurred due to food material ( *n* = 8), an acute angulation causing a dynamic obstruction ( *n* = 5), and tissue ingrowth ( *n* = 2). Stent obstruction was managed by cleaning out food from within the LAMS and/or dilating the LAMS ( *n* = 5), placing a nested LAMS or tubular metal stent ( *n* = 4), placing coaxial double-pigtail plastic stents ( *n* = 3), and removing the existing LAMS and placing a new LAMS ( *n* = 3). The 4 cases of bleeding consisted of two intraabdominal bleeds and two intraluminal bleeds. All were managed conservatively.

All four cases of perforation occurred during the procedure secondary to stent misdeployment. Three were gastric perforations without jejunal perforations (Type I), and one combined gastric and jejunal perforation with the proximal flange of the LAMS anchored on the gastric side (Type II). Two Type I and the Type II perforations were salvaged immediately by using an existing guidewire to place a new LAMS, while the other Type I perforation was managed by closing the gastric defect with an over-the-scope clipping device and performing EUS-GJ at another site. All four patients received broad spectrum antibiotics following the procedure.

## Discussion

In this study examining outcomes of EUS-GE, EUS-GJ, and EUS-GD had excellent clinical success rates, suggesting that both duodenal and jejunal access can achieve the goal of symptom palliation and allow patients to resume oral intake. There was no significant difference with respect to overall AE rate, unplanned reintervention, 30-day mortality, or mean BMI-change 4 weeks postprocedure. These findings have important clinical implications as EUS-GE is more commonly performed for the management of GOO.

The most common AEs were obstruction and perforation. A notable stent obstruction rate of 12% was likely due to several factors. Both obstruction cases in the EUS-GD group were due to an acute angulation causing dynamic obstruction, potentially representing a disadvantage of placing a stent into the more perpendicularly oriented duodenum. By contrast, stent obstruction due to food debris or tissue ingrowth may reflect the longer dwell time for these LAMS as patients live longer with their underlying malignancy. Indeed, the median time to obstruction in our study was 281 days. Adhering to a strict soft diet can be challenging for patients in the long term and lead to fibrous material occluding the stent. Additionally, the silicon coating of the stents can degrade over time ultimately resulting in tissue ingrowth. 11 Fortunately, repeat obstruction was managed endoscopically by re-establishing patency of the LAMS lumen, replacing the LAMS, placing a nested LAMS, or placing coaxial stents within the LAMS.

The misdeployment and perforation rates in our study were also lower than what has previously been reported in retrospective studies. 9 12 All perforations were all managed endoscopically. This likely represents the growing expertise of the performing endoscopists at our high-volume center. One notable finding was that all cases of perforation occurred in the EUS-GJ group, with none in the EUS-GD group. The fixed retroperitoneal location and perpendicular orientation of a gastroduodenal anastomosis may offer a more stable trajectory for LAMS placement. The small number of perforation events and lack of statistical significance, however, limit any definitive conclusions. In this study, 30-day mortality was over 20%, likely reflecting the significant burden of malignant disease in our patient population as there were no deaths directly attributable to the procedure. The relatively small sample size precludes any assessment of whether stent location influences survival.

Our study has several limitations. Due to the retrospective and single-center design, the results may be prone to selection and misclassification bias, missing data, heterogenous follow-up duration, unmeasured confounders, and limited generalizability. Second, the retrospective nature of our study does not capture the process by which a target loop was selected during the EUS-GE procedure, as finding a suitable target can be variably challenging. Third, increasing experience of operators over the course of the study and variable involvement of fellows were not formally assessed and may introduce bias into the analysis. Fourth, the two groups were not balanced, with nearly three times as many patients undergoing EUS-GJ compared to EUS-GD, and a baseline difference in BMI. This imbalance may have influenced outcomes. Despite these limitations, the results overall support the effectiveness of both EUS-GJ and EUS-GD.

Endoscopists who perform EUS-GE must be cognizant of the benefits and drawbacks of the location of stent placement. For example, an LAMS placed in the duodenum near the site of obstruction may be more prone to repeat obstruction from tumor ingrowth. In contrast, an LAMS placed in the duodenum may be utilized to access the major papilla and perform biliary intervention. 13 Endoscopists should thus consider both the safety and technical feasibility of the target small bowel and the longer-term implications to best be able to provide symptomatic relief and improve quality of life.
